# The burden of pneumoconiosis in China: an analysis from the Global Burden of Disease Study 2019

**DOI:** 10.1186/s12889-022-13541-x

**Published:** 2022-06-03

**Authors:** Jie Li, Peng Yin, Haidong Wang, Lijun Wang, Jinling You, Jiangmei Liu, Yunning Liu, Wei Wang, Xiao Zhang, Piye Niu, Maigeng Zhou

**Affiliations:** 1 School of Public Health and Beijing Key Laboratory of Environmental Toxicology, Capital Medical University, 10 Xitoutiao Road, Fengtai District, Beijing, 100069 China; 2 National Center for Chronic and Noncommunicable Disease Control and Prevention, Chinese Center for Disease Control and Prevention, 27 Nanwei Road, Xicheng District, Beijing, 100050 China; 3 Institute for Health Metrics and Evaluation, University of Washington, Seattle, WA USA

**Keywords:** Pneumoconiosis, Silicosis, Death, DALY, Disease burden

## Abstract

**Background:**

Pneumoconiosis refers to a class of serious diseases threatening the health of workers exposed to coal or silicosis dust. However, the burden of pneumoconiosis is unavailable in China.

**Methods:**

Incident cases, deaths, and disability-adjusted life years (DALYs) from pneumoconiosis and its subtypes in China were estimated from the Global Burden of Disease Study 2019 using a Bayesian meta-regression method. The trend of the burden from pneumoconiosis was analyzed using percentage change and annualized rate of change (ARC) during the period 1990–2019. The relationship between subnational socio-demographic index (SDI) and the ARC of age-standardised death rate was measured using Spearman’s Rank-Order Correlation.

**Results:**

In 2019, there were 136.8 (95% uncertainty interval [UI] 113.7–162.5) thousand new cases, 10.2 (8.1–13.6) thousand deaths, and 608.7 (473.6–779.4) thousand DALYs from pneumoconiosis in China. Of the global burdens from pneumoconiosis, more than 60% were in China. Both the total number of new cases and DALYs from pneumoconiosis was keeping increasing from 1990 to 2019. In contrast, the age-standardised incidence, death, and DALY rates from pneumoconiosis and its subtypes, except for the age-standardised incidence rate of silicosis, and age-standardised death rate of asbestosis, experienced a significant decline during the same period. The subnational age-standardised death rates were higher in western China than in eastern China. Meanwhile, the subnational ARC of age-standardised death rates due to pneumoconiosis and its subtypes were significantly negatively correlated with SDI in 2019.

**Conclusion:**

China suffers the largest health loss from pneumoconiosis in the world. Reducing the burden of pneumoconiosis is still an urgent task in China.

**Supplementary Information:**

The online version contains supplementary material available at 10.1186/s12889-022-13541-x.

## Background

Pneumoconiosis is a major public concern around the world with 0.20 (0.17–0.23) million new cases and 0.92 (0.76–1.12) million disability-adjusted life years (DALYs) in 2019 [1]. Pneumoconiosis is caused by inhalation and the retention of dust and fibers in the lung. According to types of occupational dust inhaled, pneumoconiosis is classified into asbestosis, silicosis, coal worker pneumoconiosis (CWP), and other pneumoconiosis [2]. Pneumoconiosis has a long latency, and workers may develop clinical symptoms many years after exposure to casual dust. It can also lead to a series of potentially fatal outcomes, including reduced pulmonary function, progressive massive fibrosis, disability, and premature death. There is currently no cure for pneumoconiosis and most medical treatments can only decrease further lung damage and symptoms, which highlights the importance of prevention.

China is the world’s largest labor market with more than 775 million working population [3]. According to data revealed by the National Health Commission of China, China had 15,898 new cases of pneumoconiosis in 2019, which accounted for more than 80% of the total occupational diseases that year [4]. However, due to the low coverage of occupational health examinations and strict diagnostic criteria of pneumoconiosis, the actual number of cases is likely to have been underestimated [5]. To understand the gap between the actual number and diagnosed cases, a systematic analysis of the burden due to pneumoconiosis and its trend over time is needed in China.

In this study, temporal changes in the burden of pneumoconiosis and its four major subtypes, by age and sex, during 1990–2019 in China were reported based on data from the Global Burden of Diseases 2019. The correlations between subnational socio-demographic index (SDI) and annualized rate of change (ARC) of age-standardised death rate were also determined, which hopefully will serve as a reference for policymakers to improve health.

## Methods

### Study data

National and subnational data on incidence, deaths, DALYs and the corresponding age-standardised rates of pneumoconiosis during 1990–2019 were obtained from GBD 2019 [1]. According to the International Classification of Diseases 10^th^ revision (ICD 10), pneumoconiosis (codes: J60-J65.0, J92.0) was categorized into asbestosis (codes: J61-J61.0, J92.0), CWP (codes: J60-J60.0), silicosis (codes: J62-J62.9), and other pneumoconiosis (codes: J63-J65.0). Socio-demographic index (SDI) is a composite indicator that covers income per capita, average years of educational attainment, and fertility rate in females under the age of 25 years [1]. The range of SDI was 0 to 1.

The data used to estimate mortality and disease burden of pneumoconiosis included vital registration, mortality surveillance data in China, and systematic reviews. The disease burden from pneumoconiosis was estimated separately for males and females, and also for different age groups (15–80 + years). The three levels of covariates were used in the pneumoconiosis models [1]. Level 1 covariates included asbestos consumption per capita, coal production per capita, gold production per capita, summary exposure value for occupational asbestos, occupational beryllium, and occupational silica. Level 2 covariates included smoking prevalence, indoor air pollution from all cooking fuels, 5-years cumulative cigarettes, and healthcare access and quality index. Level 3 covariates included education years per capita and SDI. All asbestos, coal, and gold covariates are included in the total pneumoconiosis model and separately used in the asbestosis model, CWP model, and silicosis model, respectively.

### Statistical analyses

The approaches and frameworks used to estimate incidence, deaths, and DALYs for pneumoconiosis have been reported previously [1, 6]. In brief, after bias adjustments via a meta-regression-Bayesian, regularised, trimmed (MR-BRT) model to allow a direct comparison between different study designs and cases, the metrics were estimated by age and sex by means of a Bayesian meta-regression tool with DisMod-MR 2.1. Prior settings of zero remission were employed for all the subtypes of pneumoconiosis. Additionally, GBD assumes that there is no incidence of pneumoconiosis before the age of 15. Meanwhile, a predictive covariate on healthcare access and quality was included in the model. Years of life lost (YLLs) were calculated by multiplying deaths by standard life expectancy at each age range. Years lived with disability (YLDs) were calculated by multiplying prevalence by disability weights. DALYs were the sum of YLLs and YLDs for each age‐sex‐location. Uncertainty intervals (UIs) for point estimation are produced by using the 25th and 975th of 1000 draw values of the posterior distribution. The percentage change is used as a summary of the age-standardised rate trend over a prespecified interval. ARC was an average summary of age-standardised rate trends over specific periods, which was the difference between the natural log of the values at the start year and the end year divided by the number of years in the interval [1]. Spearman’s correlation was employed to determine the relationships between SDI and ARC of age-standardised death rates at the subnational level.

## Results

**Fig. 1:**
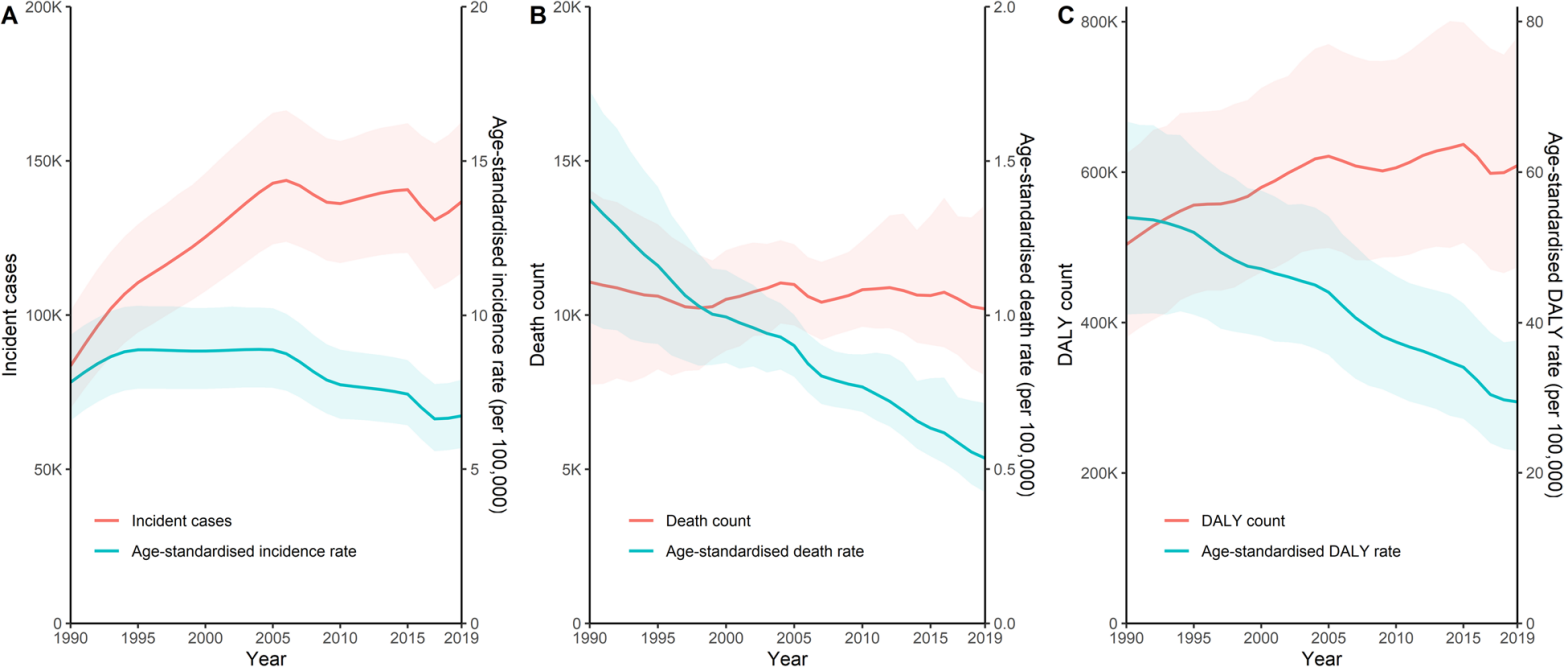
Trend of incident cases, deaths, and DALYs and corresponding age-standardised rate of pneumoconiosis in China, 1990–2019. Shaded area indicates 95% uncertainty intervals. DALY = disability-adjusted life-year

Among the total global burdens due to pneumoconiosis, 68.7% of new cases, 44.3% of deaths, and 66.2% of DALYs were in China. As shown in Table 1, new cases of pneumoconiosis grew from 83.6 (69.7–101.4) thousand cases in 1990 to 136.8 (113.7–162.5) thousand cases in 2019, which showed an increase of 63.7% (40.5%-86.7%). As shown in Fig. 1A, new cases of pneumoconiosis reached the zenith in 2006 (143.7 [123.8–166.4] thousand cases). In comparison, deaths from pneumoconiosis remained almost constant (Fig. 1B), whereas DALYs due to pneumoconiosis increased by 20.8% (-5.6%-64.0%) from 503.8 (380.5–624.5) thousand years in 1990 to 608.7 (473.6–779.4) thousand years in 2019 (Table 1 and Fig. 1C).

**Table 1:** Incident cases, Deaths, and DALYs of pneumoconiosis in China, 1990–2019

	1990	2019	Percentage change (%)
**Incident cases**			
Pneumoconiosis	83,551 (69,733–101,410)	136,755 (113,659–162,518)	63.68 (40.45–86.65)
Silicosis	68,236 (54,374–86,422)	120,775 (98,044–145,939)	76.99 (48.16–105.86)
Asbestosis	5513 (3746–7788)	4065 (2903–5337)	-26.26 (-45.01–-0.68)
CWP	5450 (4419–6665)	4974 (3976–6263)	-8.74 (-21.52–4.32)
Other pneumoconiosis	4351 (3331–5640)	6941 (5272–8870)	59.52 (42.02–78.20)
**Deaths**			
Pneumoconiosis	11,070 (7744–14,056)	10,201 (8050–13,567)	-7.85 (-37.96–60.09)
Silicosis	8401 (5612–11,032)	7746 (5939–10,860)	-7.80 (-39.61–71.42)
Asbestosis	186 (117–289)	239 (177–391)	28.53 (-25.14–145.13)
CWP	1661 (542–2518)	1302 (708–2209)	-21.62 (-54.23–88.07)
Other pneumoconiosis	821 (479–1465)	913 (655–1585)	11.27 (-37.44–104.79)
**DALYs**			
Pneumoconiosis	503,828 (380,498–624,469)	608,694 (473,558–779,381)	20.81 (-5.58–63.97)
Silicosis	402,187 (298,328–513,715)	519,697 (391,700–677,968)	29.22 (-0.93–75.88)
Asbestosis	6911 (4649–10,198)	6736 (5138–10,767)	-2.53 (-38.73–71.11)
CWP	57,207 (26,316–81,536)	41,359 (28,064–62,581)	-27.70 (-52.64–35.38)
Other pneumoconiosis	37,523 (24,113–58,935)	40,901 (30,772–61,648)	9.00 (-30.31–67.75)

*DALY* Disability-adjusted Life-year, *CWP* Coal Worker Pneumoconiosis

For different subtypes, silicosis was the leading cause of burden from pneumoconiosis, followed by CWP, other pneumoconiosis, and asbestosis (Table 1). Silicosis accounted for 88.3% (67.4%-99.3%) of new cases, 75.9% (51.6%-98.2%) of deaths, 85.4% (58.5%-99.1%) of DALYs from pneumoconiosis in China in 2019. Asbestosis took up the lowest proportion among subtypes of pneumoconiosis, accounting for 3.0% (2.0%-4.1%) of new cases, 2.3% (1.6%-4.0%) of deaths, and 1.1% (0.8%-1.9%) of DALYs from pneumoconiosis in China in 2019.

Table S1 and Table 2 present the crude and age-standardised incidence, death, and DALY rates due to pneumoconiosis in China, respectively. The crude incidence, death, and DALY rate of pneumoconiosis in China in 2019 were 9.6 (8.0–11.4), 0.7 (0.6–1.0), and 42.8 (33.3–54.8) per 100,000, respectively. From 1990 to 2019, the crude incidence rates of pneumoconiosis, silicosis, and other pneumoconiosis increased by 36.2% (16.9%-55.3%), 47.3% (23.3%-71.3%), and 32.8% (18.2%-48.3%), respectively. Almost all the age-standardised incidence, death, and DALY rates of pneumoconiosis and its subtypes significantly declined from 1990 to 2019, except for the age-standardised incidence rate of silicosis, and age-standardised death rate of asbestosis. It’s worth noting that the age-standardised incidence rate of pneumoconiosis maintained at a high level during 1994–2006 before it showed a fluctuating downward trend (Fig. 1A).

**Table 2:** The age-standardised incidence, death, and DALY rate of pneumoconiosis (per 100,000) in China, 1990–2019

	1990	2019	Percentage change (%)
**Age-standardised incidence rate**			
Pneumoconiosis	7.82 (6.58–9.36)	6.73 (5.69–7.90)	-13.92 (-22.79– -6.05)
Silicosis	6.34 (5.07–7.86)	5.92 (4.87–7.07)	-6.71 (-18.16–3.51)
Asbestosis	0.51 (0.36–0.71)	0.21 (0.15–0.27)	-58.48 (-66.99– -47.41)
CWP	0.52 (0.42–0.64)	0.25 (0.21–0.31)	-51.18 (-55.21– -46.88)
Other pneumoconiosis	0.45 (0.34–0.57)	0.35 (0.27–0.44)	-22.03 (-27.44– -16.36)
**Age-standardised death rate**			
Pneumoconiosis	1.37 (0.98–1.73)	0.54 (0.43–0.71)	-61.01 (-73.32– -33.15)
Silicosis	1.04 (0.68–1.36)	0.40 (0.31–0.56)	-61.18 (-74.34– -27.21)
Asbestosis	0.02 (0.02–0.04)	0.01 (0.01–0.02)	-45.20 (-66.86–4.04)
CWP	0.21 (0.08–0.32)	0.07 (0.04–0.12)	-67.21 (-80.29– -23.14)
Other pneumoconiosis	0.09 (0.06–0.17)	0.05 (0.04–0.08)	-49.11 (-70.82– -7.10)
**Age-standardised DALY rate**			
Pneumoconiosis	53.98 (41.08–66.73)	29.45 (22.95–37.61)	-45.44 (-57.36– -26.34)
Silicosis	43.21 (32.28–55.06)	24.97 (18.90–32.51)	-42.21 (-55.47– -21.16)
Asbestosis	0.70 (0.47–1.03)	0.35 (0.27–0.56)	-49.81 (-68.16– -12.55)
CWP	6.26 (2.92–8.74)	2.06 (1.39–3.09)	-67.10 (-78.29– -37.71)
Other pneumoconiosis	3.82 (2.52–5.88)	2.07 (1.56–3.12)	-45.71 (-64.48– -18.27)

*DALY* Disability-adjusted Life-year, *CWP* Coal worker pneumoconiosis

**Table 3:** Age-standardised incidence, death, and DALY rate of pneumoconiosis (per 100,000) by sex in China, 2019

	Age-standardised incidence rate	Age-standardised death rate	Age-standardised DALY rate
**Males**			
Pneumoconiosis	12.51 (10.49–14.72)	1.16 (0.91–1.56)	57.92 (45.02–74.00)
Silicosis	11.23 (9.20–13.44)	0.89 (0.69–1.25)	49.92 (37.96–65.07)
Asbestosis	0.30 (0.21–0.38)	0.02 (0.01–0.04)	0.53 (0.37–0.93)
CWP	0.44 (0.36–0.54)	0.16 (0.08–0.26)	3.99 (2.57–6.15)
Other pneumoconiosis	0.55 (0.43–0.69)	0.09 (0.06–0.16)	3.48 (2.56–5.42)
**Females**			
Pneumoconiosis	0.89 (0.74–1.08)	0.06 (0.04–0.08)	2.92 (2.24–3.80)
Silicosis	0.52 (0.38–0.69)	0.03 (0.02–0.04)	1.64 (1.18–2.26)
Asbestosis	0.14 (0.09–0.18)	0.01 (0.01–0.01)	0.19 (0.13–0.31)
CWP	0.08 (0.06–0.10)	0.01 (0.00–0.01)	0.33 (0.23–0.45)
Other pneumoconiosis	0.16 (0.13–0.21)	0.01 (0.01–0.02)	0.76 (0.55–1.04)

*DALY* Disability-adjusted Life-year, *CWP* Coal Worker Pneumoconiosis

**Fig. 2:**
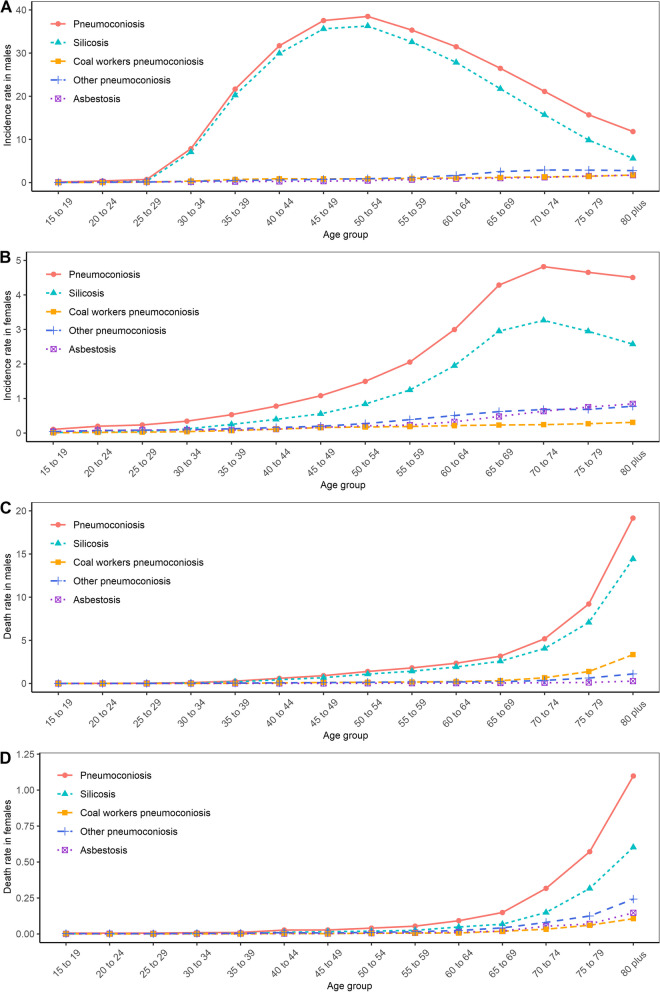
Sex- and age-specific incidence, death rate of pneumoconiosis (per 100,000) and its subtypes in China, 2019. **A** Age-specific incidence rate in males, **B** age-specific incidence rate in females, **C** age-specific death rate in males, and **D** age-specific death rate in females

New cases, deaths, and DALYs due to pneumoconiosis in males accounted for 93.3%, 94.5%, and 95.2% of the corresponding total numbers in 2019 (Table S2). Table 3 shows the age-specific incidence, death rates due to pneumoconiosis by sex in 2019. As shown in Fig. 2A and 2B, the age-specific incidence rate of pneumoconiosis and silicosis increased rapidly after the age of 25, reaching the zenith in the age group 50–54 in males, and the age group 70-74 in females. The death rate of pneumoconiosis and silicosis rose sharply after the age of 35, reaching the highest in the age group 80 or older in both sexes (Fig. 2C and 2D). The peak DALY rate of pneumoconiosis and its subtypes was observed in the age group 80 or older in both males and females (Fig. S1).

**Fig. 3:**
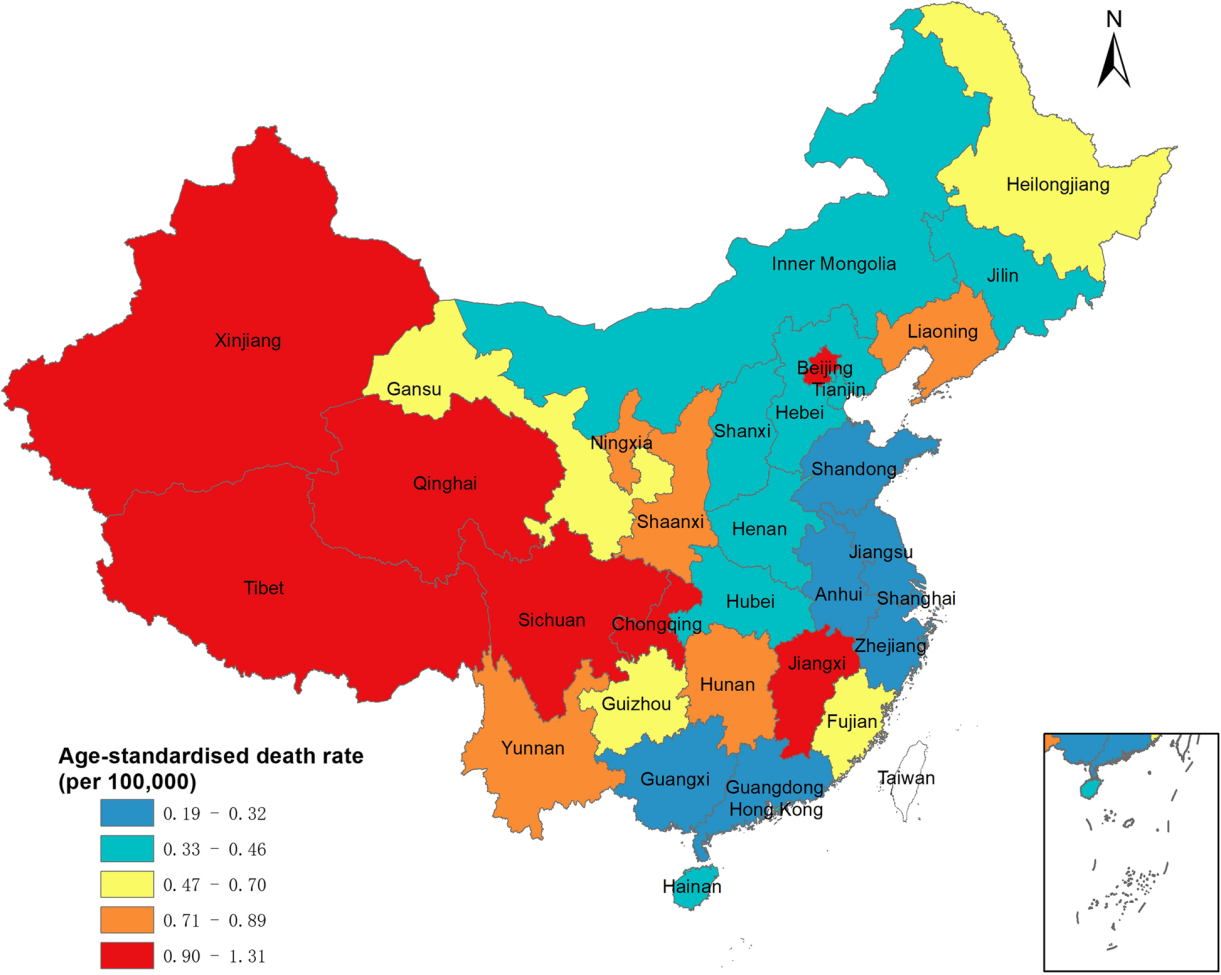
Age-standardised death rate (per 100,000) of pneumoconiosis in the provinces of China, 2019

Figure 3 presents the subnational estimate of age-standardised death rates. Western China, especially Xinjiang, Chongqing, and Qinghai, reached the highest age-standardised death rate from pneumoconiosis in 2019, whereas eastern China including Shanghai, Guangdong, and Shandong had lower age-standardised death rates due to pneumoconiosis.

Subnational ARC of the age-standardised death rate due to pneumoconiosis from 1990 to 2019 was negatively correlated with SDI in 2019 (*r* = -0.591, *p* < 0.001). As for the different subtypes, significant negative correlations were observed between SDI and ARC of age-standardised death rate due to silicosis (*r* = -0.709, *p* < 0.001), CWP (*r* = -0.469, *p* = 0.007), asbestosis (*r* = -0.800, *p* < 0.001), and other pneumoconiosis (*r* = -0.714, *p* < 0.001) from 1990 to 2019.

## Discussion

Accounting for 18.5% of the world’s population, China has a disproportionately high proportion (66.2%) of the global DALYs due to pneumoconiosis. From 1990 to 2019, the counts of incidence and DALYs due to pneumoconiosis kept increasing, whereas the age-standardised incidence, death, and DALY rate from pneumoconiosis decreased by more than 14%. The correlations between subnational SDI and ARC of the age-standardised death rate due to pneumoconiosis in 2019 were significantly negative.

Pneumoconiosis is the leading occupational disease and the third cause of burden due to chronic respiratory diseases in China. Among the estimated 775 million workers in China, more than 20 million workers were exposed to occupational risk factors in 2019 [7]. The estimated incidence of pneumoconiosis was 136.8 thousand cases in GBD 2019, compared to 15.9 thousand new cases as reported by the government in 2019, China [4]. The underestimate of government report could be attributed to the following reasons. First, large numbers of cases were unreported in small informal workshops or township enterprises with dangerous working conditions [5]. Second, the government report was based on the diagnosed cases which had to meet strict diagnostic criteria of pneumoconiosis. Moreover, the mobility of migrant workers and the long latency of pneumoconiosis caused missing reports. There are about 280 million migrant workers in China [8]. Those migrant workers are likely to be employed in mines, the construction industry, and the manufacturing industry. Their mobility gives rise to loose occupational surveillance. Most risk factors for occupational diseases especially pneumoconiosis are preventable, and there is an urgent need for migrant workers to have more strict occupational surveillance, vocational training, and safety education.

Among four subtypes of pneumoconiosis, silicosis was the leading cause of disease burden from pneumoconiosis, followed by CWP, other pneumoconiosis, and asbestosis in China in 2019. As their names imply, silicosis, coal workers’ pneumoconiosis, and asbestosis are caused by inhaling silica dust, coal mine dust, and asbestos fibers, respectively. As the burden of pneumoconiosis was 100% attributable to occupational exposure, our findings are consistent with previous studies that occupational silica is still the leading risk factor for pneumoconiosis [9–11]. Other pneumoconiosis includes berylliosis, aluminous, siderosis, stannosis, and baritosis that result from inhalation of beryllium, aluminum, iron, tin fume, and barium sulfate particles, respectively [12]. From 1990 to 2019, the new cases of pneumoconiosis, silicosis, and other pneumoconiosis increased by more than 50%. After removal of the effects from population growth, the crude rates of incidence due to pneumoconiosis, silicosis, and other pneumoconiosis were still significantly higher in 2019 than in 1990. However, after further removal of the effects from aging, all the age-standardised incidence rates significantly declined except for silicosis. The age-standardised incidence rate of silicosis remained stable from 1990 to 2019, suggesting the rising trend of new cases due to silicosis showed no signs of abating in China. There is an urgent need for more protection strategies and policy support in order to reduce silicosis.

Age-standardised death and DALY rates of pneumoconiosis and its subtypes decreased by more than 40%, which likely was a result of the persistent efforts by the Chinese government. The greatest number of new cases from pneumoconiosis was observed in 2006, and the age-standardized incidence rate of pneumoconiosis started to drop from 2006 when China began to take strong supervision measures. In 2006, China established the Network Direct Report System of Occupational diseases to monitor occupational diseases. A series of government documents, including the Plan for a Health China 2030 and the 13th Five-Year Plan for Occupational Health Hazard Prevention and Control, have been released to improve occupational health in recent years in China [13]. For example, 95% of workers exposed to occupational hazards for pneumoconiosis must go through regular health checks by 2020. Moreover, China revised and improved the law of occupational disease prevention and control in 2016. The decreased burden of pneumoconiosis could also be attributed to intensified regulatory supervision, expanded medical accessibility, and improved medical conditions.

The new cases of asbestosis remained high in China in 2019. The risk factor of asbestosis is occupational exposure to asbestos fibers which is listed as carcinogenic to humans (Group 1) by the International Agency for Research on Cancer [14]. The use of asbestos has been banned or strictly regulated in countries such as the United States, Australia, and many in western Europe have since the 1970s and 1980s [15]. A few countries, especially Russia, Brazil, China, and India, have continued to use large quantities of asbestos [16]. China produced about 125,000 tons of asbestos, accounting for 11.4% of global total production in 2019 [17]. We also observed that summary exposure values (SEVs) of most occupational carcinogens especially asbestos have increased in the past three decades [7]. Asbestos exposure usually occurred in construction and shipyards [16]. In order to prevent the public health crisis triggered by the use of asbestos, we must reduce, or even ban asbestos use. At the same time, we need to develop replacement materials and take necessary measures that can help to prevent asbestosis and other related diseases.

Our result showed the new cases, deaths, and DALYs due to pneumoconiosis in males accounted for about 95% of the corresponding total numbers in 2019, which was consistent with previous reports [18]. The predominance of males as observed in incident cases, deaths , and DALYs of pneumoconiosis might be explained by the following reasons. Dust inhalation in the coal, metal, and construction industries is the main cause of pneumoconiosis, and workers in those industries are predominantly men. Therefore, male workers were exposed to relatively higher dust levels than females. In addition, a higher level of smoking in male workers might also have contributed to the male predominance in the incidence of pneumoconiosis. Cigarette smoking can accelerate the onset of silicosis dust by destroying the bronchi’s ciliated epithelium and decreasing the pulmonary clearance capacity [19]. This phenomenon has also been observed in other studies. For instance, according to a recent study, the incidence of silicosis in men (17%) was significantly higher than in women (4%) at the same average cumulative silica dust exposure level [19]. The higher mortality and DALY rate in males than in females were mainly related to the higher incidence of pneumoconiosis in the former. The peak age-specific incidence rate of pneumoconiosis was observed in the age group 50–54 in males, which was inconsistent with the age group 65–69 reported in Great Britain during 1996–2014 [18]. The possible explanations included the difference between the study periods, research area, and disease classification. Moreover, recent studies reported shorter latency periods of silicosis are observed in stonecutters [20, 21].

The subnational results showed the age-standardised death rate of pneumoconiosis in western China was higher than that in the eastern coastal area, which might have been a result of the difference in economic development level. As SDI, a key factor affecting the socio-economic development of a specific region, was used to explain the regional variations, the relationship between SDI and ARC of the age-standardised death rate due to pneumoconiosis was further analyzed [22]. The results showed subnational SDI was negatively correlated with the ARC of the age-standardised death rate due to pneumoconiosis and its subtypes, which was in accordance with previous studies [10, 23]. The spatial inequality of socioeconomic level and medical services have impacts on the burden of occupational diseases [7]. Compared to less developed areas, especially western China, developed areas such as the eastern coastal area in China own more institutions certificated to diagnose the occupational disease and have a stronger regulatory system. Western China is thus in urgent need of adequate supervision and medical services of occupational diseases.

Although China has been making ceaseless efforts to prevent and control occupational diseases, especially pneumoconiosis, the challenges remain huge as shown in our study. In order to reduce the incidence and burden due to pneumoconiosis in China, much work is needed to improve the workplace environment, provide personal protective equipment, guide employers to standardized work safety protocols, perfect pneumoconiosis surveillance systems, and increase the intensity of regulatory supervision [13]. Additionally, research on technical support for prevention, effective hazards monitoring, frontier fundamental research, key techniques of diagnosis and treatment of pneumoconiosis should be strongly encouraged.

Our study provides a comprehensive summary of incidence, mortality, and DALY rates from pneumoconiosis and its subtypes from 1990 to 2019 in China and represents the most up-to-date information on burdens due to pneumoconiosis. Meanwhile, the standardized estimation method employed by GBD allows us to make sub-national comparisons in China. Furthermore, the attribution of SDI is examined to explain the variations of annualized change rate in burden of pneumoconiosis.

However, this study is still limited in several ways. First, national and subnational data are unavailable for the occupational exposure levels of dust and fibers in China. The main covariates in pneumoconiosis models in GBD 2019 were asbestos consumption per capita, coal production per capita, gold production per capita, summary exposure value for occupational asbestos, occupational beryllium, and occupational silica [1]. The consumption of other dust containing antimony, barium, graphite, and iron was not taken into consideration in the models, which may lead to an underestimate of the burden of pneumoconiosis. Second, occupational epidemiological studies in China are scarce compared with developed countries. GBD employs covariates from developed countries to estimate the burden of occupational diseases which may cause uncertainties in the results [1]. Third, GBD only includes disabilities associated with the most common sequelae of occupational exposure, but not mental disorders. Furthermore, the joint effects of co-exposure to dust and fibers may lead to misclassification of pneumoconiosis. About 23% of pneumoconiosis deaths and DALYs from ‘Other pneumoconiosis’ were actually due to the three main pneumoconiosis (silicosis, asbestosis, or CWP) [23]. Further work, especially the national occupational investment and comprehensive occupational health monitoring, is expected to expand accessible resources and improve accuracy of estimates in China.

## Conclusion

Our findings show that two-thirds of global health loss from pneumoconiosis occur in China. Prevention and control of pneumoconiosis is still serious health and social issue in China. The heterogeneity between different areas of China highlights the urgent need for preferential policy to less developed regions.

## Supplementary Information

**Additional file 1:** **Table S1.** Crude incidence, death, and DALY rate of pneumoconiosis (per 100,000) in China, 1990-2019. **Table S2.** Incident cases, deaths, and DALYs of pneumoconiosis by sex in China, 2019. **Figure S1.** Sex- and age-specific DALY rate of pneumoconiosis (per 100,000) and its subtypes in China, 2019. (A) Males and (B) females. 

## Data Availability

The datasets used during the current study are available online on the official website of the Institute of Health Metrics and Evaluation (http://ghdx.healthdata.org/gbd-results-tool).

## Abbreviations

ARC: Annualized rate of change
CWP: Coal worker pneumoconiosis
DALYs: Disability-adjusted life years
GBD: Global Burden of Diseases Study
ICD: International Classification of Diseases
SDI: Socio-demographic index
SEVs: Summary exposure values
UI: Uncertainty interval
YLDs: Years lived with disability
YLLs: Years of life lost
