# Supplementary material for: The burden of pneumoconiosis in China: an analysis from the Global Burden of Disease Study 2019
Source: BMC Public Health. 2022 Jun 3;22:1114. doi: 10.1186/s12889-022-13541-x (PMC9166455; doi:10.1186/s12889-022-13541-x)
Supplement: Supplementary file 1 — Additional file 1: Table S1. Crude incidence, death, and DALY rate of pneumoconiosis (per 100,000) in China, 1990-2019. Table S2. Incident cases, deaths, and DALYs of pneumoconiosis by sex in China, 2019. Figure S1. Sex- and age-specific DALY rate of pneumoconiosis (per 100,000) and its subtypes in China, 2019. (A) Males and (B) females. [file 12889_2022_13541_MOESM1_ESM.docx]

**Supplementary material**

**The Burden of Pneumoconiosis in China: An Analysis from the Global Burden of Disease Study 2019**

Jie Li, Peng Yin, Haidong Wang, Lijun Wang, Jinling You, Jiangmei Liu, Yunning Liu, Wei Wang, Xiao Zhang, Piye Niu*, Maigeng Zhou*

Table S1. Crude incidence, death, and DALY rate of pneumoconiosis (per 100,000) in China, 1990-2019

Table S2. Incident cases, deaths, and DALYs of pneumoconiosis by sex in China, 2019

Figure S1. Sex- and age-specific DALY rate of pneumoconiosis (per 100,000) and its subtypes in China, 2019. (A) Males and (B) females.

| Table S1. Crude incidence, death, and DALY rate of pneumoconiosis (per 100,000) in China, 1990-2019 | | | |
| --- | --- | --- | --- |
|  | 1990 | 2019 | Percentage change (%) |
| **Incidence** |  |  |  |
| Pneumoconiosis | 7.06 (5.89-8.57) | 9.61 (7.99-11.43) | 36.21 (16.88-55.33) |
| Silicosis | 5.76 (4.59-7.30) | 8.49 (6.89-10.26) | 47.30 (23.30-71.31) |
| Asbestosis | 0.47 (0.32-0.66) | 0.29 (0.20-0.38) | -38.63 (-54.24--17.35) |
| CWP | 0.46 (0.37-0.56) | 0.35 (0.28-0.44) | -24.06 (-34.69--13.19) |
| Other pneumoconiosis | 0.37 (0.28-0.48) | 0.49 (0.37-0.62) | 32.75 (18.19-48.30) |
| **Death** |  |  |  |
| Pneumoconiosis | 0.94 (0.65-1.19) | 0.72 (0.57-0.95) | -23.31 (-48.37-33.22) |
| Silicosis | 0.71 (0.47-0.93) | 0.54 (0.42-0.76) | -23.27 (-49.74-42.66) |
| Asbestosis | 0.02 (0.01-0.02) | 0.02 (0.01-0.03) | 6.96 (-37.70-104.00) |
| CWP | 0.14 (0.05-0.21) | 0.09 (0.05-0.16) | -34.78 (-61.91-56.51) |
| Other pneumoconiosis | 0.07 (0.04-0.12) | 0.06 (0.05-0.11) | -7.40 (-47.93-70.42) |
| **DALY** |  |  |  |
| Pneumoconiosis | 42.56 (32.15-52.76) | 42.79 (33.29-54.80) | 0.54 (-21.43-36.46) |
| Silicosis | 33.98 (25.20-43.40) | 36.54 (27.54-47.67) | 7.54 (-17.55-46.37) |
| Asbestosis | 0.58 (0.39-0.86) | 0.47 (0.36-0.76) | -18.89 (-49.01-42.40) |
| CWP | 4.83 (2.22-6.89) | 2.91 (1.97-4.40) | -39.83 (-60.58-12.66) |
| Other pneumoconiosis | 3.17 (2.04-4.98) | 2.88 (2.16-4.33) | -9.29 (-42.00-39.60) |
| DALY=disability-adjusted life-year; CWP=Coal worker pneumoconiosis | | | |

| Table S2. Incident cases, deaths, and DALYs of pneumoconiosis by sex in China, 2019 | | | |
| --- | --- | --- | --- |
|  | Incident cases | Deaths | DALYs |
| **Males** |  |  |  |
| Pneumoconiosis | 127648 (105380-151758) | 9642 (7442-12995) | 579259 (448047-744310) |
| Silicosis | 115336 (93287-139798) | 7460 (5677-10569) | 502928 (378572-654931) |
| Asbestosis | 2754 (1963-3643) | 163 (107-300) | 4917 (3435-8638) |
| CWP | 4214 (3393-5316) | 1245 (657-2166) | 38015 (24663-59820) |
| Other pneumoconiosis | 5344 (4023-6903) | 774 (519-1426) | 33398 (24424-53332) |
| **Females** |  |  |  |
| Pneumoconiosis | 9107 (7520-11114) | 559 (383-754) | 29435 (22555-38505) |
| Silicosis | 5439 (3991-7313) | 287 (162-391) | 16768 (12041-23072) |
| Asbestosis | 1311 (900-1797) | 76 (49-126) | 1819 (1269-2928) |
| CWP | 760 (579-972) | 57 (30-85) | 3344 (2366-4605) |
| Other pneumoconiosis | 1597 (1211-2092) | 139 (96-235) | 7503 (5424-10193) |
| DALY=disability-adjusted life-year; CWP=Coal worker pneumoconiosis | | | |


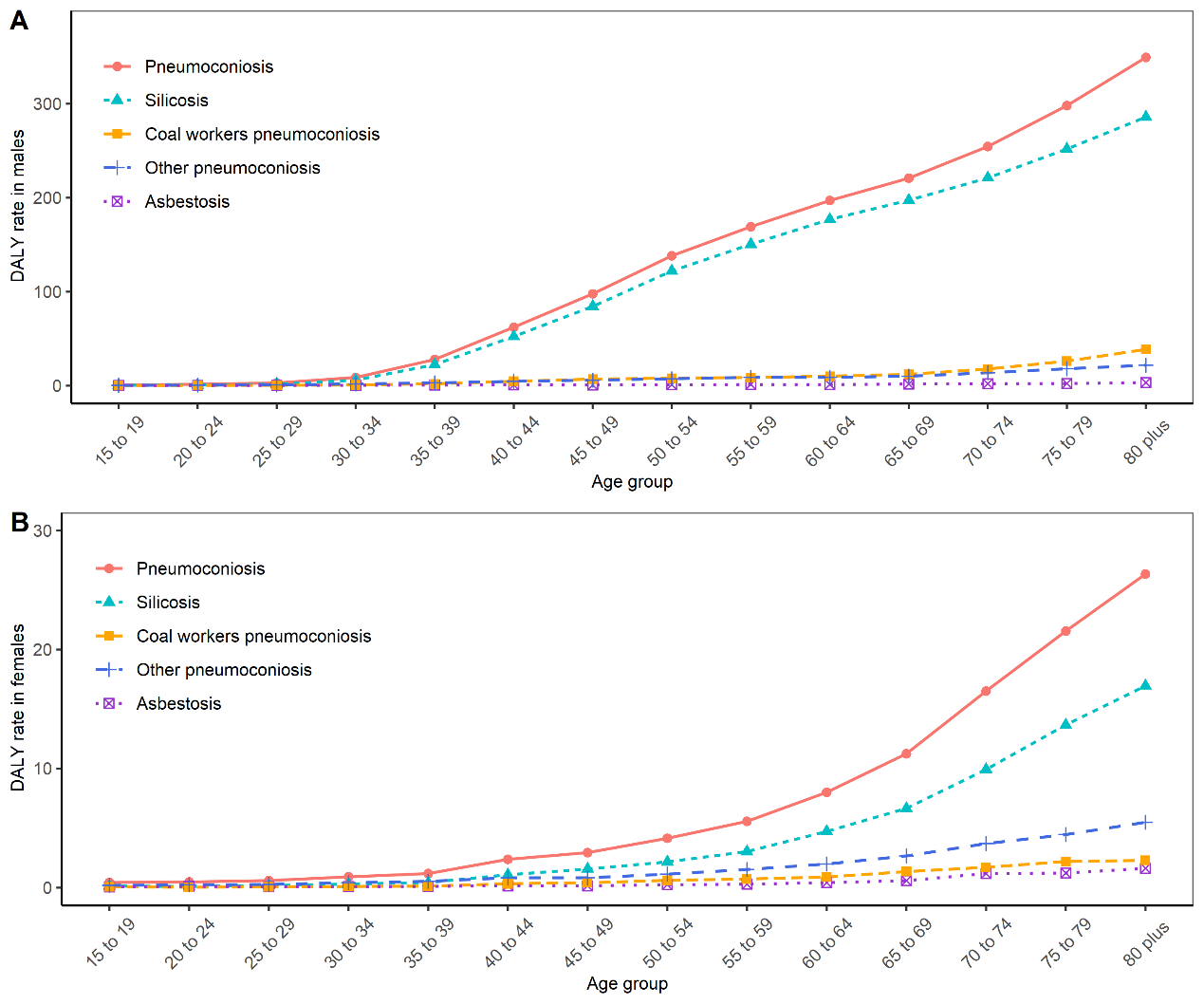
Figure S1. Sex- and age-specific DALY rate of pneumoconiosis (per 100,000) and its subtypes in China, 2019. (A) Males and (B) females.
